# Cabergoline-Induced Psychosis: A Case Report

**DOI:** 10.1192/bjo.2026.11758

**Published:** 2026-06-30

**Authors:** Aysu Ilayda Acikgoz

**Affiliations:** Hertfordshire Partnership NHS Trust, Hatfield, United Kingdom

## Abstract

**Aims:**

The majority of prolactinomas can be managed medically with dopamine agonists. These work by inhibiting the release of prolactin from the anterior pituitary, and therefore resolving hyperprolactinemia symptoms, reducing tumor size, and often restoring reproductive function. Cabergoline is the preferred dopamine agonist for the treatment of prolactinomas due to its superior efficacy compared to other dopamine agonists. Furthermore, it is generally well tolerated as compared to other dopamine agonist agents due to its more selective D2 receptor agonistic effect. It is a stimulant of dopamine receptors in the brain and it also inhibits release of prolactin by the pituitary. Additionally, it has applications in the treatment of Acromegaly, Parkinson’s disease, and Restless Leg Syndrome. Cabergoline is linked to a range of side effects, including cardiovascular, gastrointestinal, and neuropsychiatric issues. However, the literature on cabergoline-induced psychosis, particularly in individuals with no prior mental health diagnoses, is notably limited.

To our knowledge, there are very limited number of reports on Cabergoline induced psychosis especially in the context of no prior mental health disorder. Medicines and Healthcare products Regulatory Agency shares that there were 1101 total reactions to Cabergoline from 1994 until 2024 and 187 of these were psychiatric reactions. Out of these 187, there is only 5 reports of psychotic disorder and 1 report of acute psychosis.

We present a case of a 37-year-old male with no previous mental health diagnoses who developed acute psychosis following a dose increase of cabergoline for the treatment of prolactinoma. In individuals who are predisposed, cabergoline may trigger psychotic episodes; therefore, it should be used with caution and should be monitored closely.

**Methods:**

This case report is of a 37-year-old men who had no previous contact with mental health services and no previous diagnosis of mental health disorders. Apart from a period during which he was suffering from gambling addiction from 2008 until 2021. He does not use any illicit drugs. He has a family history of completed suicide. He lives with his wife and their 4-year-old daughter. He works as a warehouse operator.

The patient was diagnosed with pituitary adenoma in July 2021 after presenting with fatigue. This adenoma was found to be microadenoma and Cabergoline was started in July 2021. His wife reported some changes in his personality when he initially started cabergoline, such as fluctuating mood and irritability. The dose was stabilized at 1 mg/week up until the end of May 2024. In May 2024, MRI scan showed stable appearances in the left-sided pituitary adenoma and the dose of Cabergoline was increased to 1.5 mg three times a week due to size growth and increase in prolactin levels.

In roughly a months’ time, the patient started to become unwell which started with prominent low mood and isolation. This then evolved into excessive rumination and fixation around his work colleagues within a few days. The patient was paranoid of his colleagues, believed they were manipulating him and laughing at him, as well as believing his wife was infidelity. His wife observed him having sleeping issues and pacing up and down the house. They had a trip abroad planned during which patient became increasingly unwell. He believed people at work were spreading lies about his sexual orientation, had delusional memories and excessive worry about his health. His family seeked help from a psychiatrist who prescribed him Aripiprazole, but patient believed this medication would kill him and refused to take it. Around this time, he started experiencing paranoia about being monitored by the government and the police via hidden cameras, accused his family of trying to kill him and believed there were microchips in his phone and his brain, as well as his family, including their cat. It was at this time he stopped eating and drinking and only consumed bottled, unopened water. At this point the patient also reported olfactory (smelling a strong perfume) and auditory hallucinations (hearing people walking in the apartment at night). He developed a fear of going to the toiles, as he would see snakes on the bathroom floor. When he was watching the TV, he would see snakes and guns on the TV. He would see King Charles and Rishi Sunak on the TV and would think that they are talking about him. He thought people and cars were following him.

The patient stopped taking cabergoline by mid-July, however he was still paranoid. He accused his father of sexually abusing him. Him and his family cut their trip short and returned to the UK. On the flight back, he was very distressed as he could see vampires and dead people walking up and down the plane. After arrival, patient started to believe he had to kill his father as he was “the snake” in the family. He attempted to get a flight but did not have a ticket nor his passport as he threw it away prior to making it to the airport.

At this point, he was taken to an emergency department and community support was offered. However, due to increasing aggression towards his family, the police was called. He asked police to take him to Russia as he “needed to speak to Putin” and was placed on s136.

Following this, a MHAA was requested, and social services were involved. After the MHAA he patient was detained under section 2 of the MHA 1983. He was admitted to an inpatient unit where he was started on Olanzapine which was then switched to Haloperidol. In a weeks’ time, beginning of August, all his psychotic symptoms were resolved. However due to EPSEs, namely akathisia and acute dystonia, his Haloperidol was again switched to Aripiprazole. He did not experience any psychotic symptoms during the switch. Aripiprazole dose at this point was 10 mg PO OD. This was further reduced to 5 mg 3 months later and was discontinued after the patient`s surgery for his prolactinoma, as he is no longer able to use medication treatment for his prolactinoma. Currently, the patients is well with no mental health problems.

**Results:**

The current literature heavily focuses on exacerbation of mania and to some extent schizophrenia in patients who were started on cabergoline. However, the data is very limited on cases with no prior mental health problems. The case reports available mainly focus on these exacerbations in women and interestingly there is a very limited number of reports in men.

Therefore, this case is notable for the emergence of psychotic symptoms in a male patient with no psychiatric history following dose increase of an existing cabergoline therapy, highlighting a potential risk that is underrecognised in the current literature. While the dopaminergic mechanism of cabergoline provides a plausible theoretical explanation for the induction of psychosis, existing research places limited emphasis on this adverse effect, particularly in individuals without known vulnerability.

Our findings demonstrate that psychiatric side effects may occur following dopamine agonist therapy even in patients without pre-existing mental health conditions, reinforcing the importance of early and ongoing monitoring.

**Conclusion:**

These findings highlight that clinicians should maintain a high index of suspicion for psychiatric side effects of dopamine agonists, even in patients without prior mental illness, and emphasise the importance of close monitoring following treatment initiation or dose increase. It also suggest the value of collaborative work within different specialities, to ensure monitoring, early recognition and management of adverse effects. Reporting such reactions to relevant drug safety monitoring authorities is also essential to strengthen the evidence base and improve patient safety.

